# Isolation of a Novel *Pythium* Species, *P. thermoculicivorax*, and *Trichoderma* sp. from Natural Enzootic Mosquito Larval Infections

**DOI:** 10.3390/jof10030199

**Published:** 2024-03-05

**Authors:** Ross Joseph, Constance Darrisaw, Aaron Lloyd, David Hoel, Nemat O. Keyhani

**Affiliations:** 1 Department of Biological Sciences, University of Illinois, Chicago, IL 60607, USA; rossj@uic.edu; 2 Lee County Mosquito Control District, Lehigh Acres, FL 33971, USA; darrisaw@lcmcd.org (C.D.); lloyd@lcmcd.org (A.L.)

**Keywords:** oomycete, *Pythium*, mosquito larvae, thermotolerance, microbial pathogen, *Trichoderma*

## Abstract

Only a handful of microbial mosquito larval pathogens have been described to date. Sampling several natural enzootic infections of mosquito larvae in southwestern Florida indicated the presence of microbial pathogens capable of extensive larval mortality. A microscopic analysis of one sample site revealed extensive apparent growth of a *Pythium*-like microbe on mosquito larvae, with the highest degree of infection observed in the siphon and head regions. Structures consistent with sporangia were seen on infected insects after lactophenol blue staining, and higher-resolution scanning electron microscopy (SEM) micrographs showed sporangia and encysted zoospores targeting the head and siphon regions. The isolate was single-colony purified, and molecular identification targeting the ITS and COX1 loci coupled to phylogenetic reconstruction indicated that the isolate belonged to the *Pythium* genus but was distinct from its closest characterized species, *P. inflatum*. Morphological features were characterized, with the isolate showing rapid growth on all mycological media tested and relatively high thermotolerance, capable of robust growth at 37 °C; hence, it was designated *P. thermoculicivorax*. Sampling from a second series of natural infections of mosquito larvae resulted in the molecular identification of three *Trichoderma* isolates, one with high similarity to *T. strigosum* and the other two clustering closely with *T. asperellum*. These data highlight the occurrence of natural enzootic infections of mosquito larvae, potentially as a resource for the identification of new mosquito pathogens.

## 1. Introduction

Interest in microbial insect pathogens stems, in part, from their potential as biological agents that can be used as alternatives to chemical pesticides due to their lower environmental toxicity and decreased off-target effects [1,2,3]. Examining naturally occurring enzootic diseases, particularly of insect pests, is important for increasing our understanding of basic ecological processes, but it also represents a reservoir for the discovery of locally adapted biological agents that could potentially be exploited for pest control [4,5,6]. Mosquitoes represent a major global health threat due to the variety of disease-causing agents, ranging from viruses (e.g., dengue, yellow fever, and Zika) to parasites (e.g., malaria-causing *Plasmodium* sp.), that they vector [7,8,9]. Human and animal (livestock) cases exceed hundreds of millions per year, resulting in significant social and economic losses, with several million deaths of humans and livestock animals reported annually and even links to certain cancers suspected [10,11]. Both biological and chemical methods have been used to control mosquito populations, with the latter predominating; however, the development of populations resistant to synthetic chemical pesticides and increasing regulatory burdens have shifted some attention to the former category [12,13,14,15]. Amongst microbial biological agents, the best characterized are Ascomycete entomopathogens (e.g., *Beauveria* and *Metarhizium* sp.), some of which have been commercialized for the control of larvae and adults from a range of mosquito species, including those that have developed resistance to chemical pesticides [16,17,18,19,20,21]. In addition, several mosquito pathogens within the Oomycota, which represent a distinct phylogenetic lineage (pseudofungi and Stramenopiles) apart from the Fungal Kingdom, including a variety of plant and animal pathogens previously described [22,23,24].

Among oomycete mosquito pathogens, *Lagenidium giganteum* (Couch ex Redhead, 2015) has been widely examined as a potential mosquito larval pathogen, with a commercialized product briefly approved by the US Environmental Protection agency [25,26,27,28]. The product was subsequently deregistered due to the reporting of several cases of *L. giganteum*-mediated near-fatal infections in dogs in the southern US. Two populations of *L. giganteum* have been reported, and while both are able to infect mosquito larvae, one, labeled heat-tolerant (capable of growing at 37 °C), appears to infect mammals, and the other, heat-sensitive (incapable of growing at 37 °C), infects mosquito larvae in nature [28,29]. The characterization of additional isolates from fly, mosquito, and a dog with lagenidiosis (the former two heat-sensitive, the latter heat-tolerant) identified a new species named *L. juracya* (Vilela, Humber, Taylor, and Mend, 2019), and the dog-derived isolate was named *Paralegenidium ajellopsis* (Vilela, Humber, Taylor and Mend, 2019) [24]. *Leptolegnia chapmanii* (Seym, 1984) is another oomycete pathogen of mosquito larvae, with various life cycle parameters and histological examinations of zoospore penetration via the integument and gut described [23,30,31]. More recently, *L. chapmanii* has been shown to be compatible with the chemical insecticide diflubenzuron and with neem oil, potentially as part of an integrated pest management (IPM) strategy for larval mosquito control [32].

Several *Pythium* species have also been described as mosquito pathogens including *P. carolinianum* (Matthews, 1931), *P. guiyangense* (Su, 2006), and even isolates of the mammalian pathogen *P. insidiosum* (De Cock, Mend, Padhye, Ajello and Kaufman, 1987) [33,34,35]. Genomic sequencing and molecular studies have identified aspects of the infection mechanism and annotated a set of effector proteins in *P. guiyangense* [36,37], although these studies lag far behind those on plant pathogenic oomycetes. Here, we report the occurrence of several natural enzootic infections of mosquito larvae in the southwestern part of Florida. Microscopic and physiological analyses and the sequencing and analysis of marker genomic loci (ITS and COXI) indicated the identification of a new heat-tolerant *Pythium* species, designated *P. thermoculicivorax*, while sequencing the markers ITS, RPB2, and TEF1a revealed three mosquito pathogenic *Trichoderma* isolates, one of which partitioned with *T. strigosum* (Bissett, 1991), whereas the other two were closely related to each other and clustered with *T. asperellum* (Lieckfeldt and Nirenberg, 1999).

## 2. Materials and Methods

### 2.1. Collecting Mosquito Larvae

Larval collection was completed using a standard larval dipper (Clarke, St. Charles, IL, USA) and a fine mesh food strainer (AIYoo, Amazon.com, Seattle, WA, USA) functioning as a hand sieve. When a potential larval breeding site was found, the dipper was used to scoop up a visible group of larvae or debris in which larvae may have been hiding. The contents of the dipper were poured through the hand sieve to remove excess water. Sieved larvae were rinsed with source water and placed in a collection container. Once enough larvae were collected, a small amount of water from the larval source was added to the container to ensure larval survival during transport. Once brought into the insectary, larvae were sieved again to separate them by size and remove debris. They were rinsed with clean water and added to pans of filtered tap water. Subsets of each collected larval sample were separated and taken under a microscope to determine the presence and degree of pathogen infection. All mosquito larvae were identified by eye and then confirmed under a microscope using the method of Darsie and Ward [38]. For most samples, a record was kept of the mosquito species, the location of collection, the larval instar, and the degree of infection, if any, at the time of reception.

### 2.2. Isolation of Pythium and Trichoderma Strains

Infected mosquito larvae collected in Lee County were sealed in plastic bags half-filled with water and shipped on ice overnight to the University of Florida (main campus, Gainesville, FL, USA) for the isolation of *Pythium* and *Trichoderma* species. Upon receipt, larvae were removed from the bag and placed into a petri dish filled with sterile distilled water. To wash the larvae, the dish was swirled and then the water was removed and replaced with fresh sterile distilled water. A total of three washes were performed in this manner. Individual larvae were plated on 60 mm potato dextrose agar (PDA) (Thermo Scientific, Waltham, MA, USA) plates amended with 200 µg/mL of ampicillin and streptomycin (Sigma Aldrich, St. Louis, MO, USA) by cutting a small wedge out of the agar in the center of the plate and placing the larvae inside. Plates were incubated at room temperature (~24 °C) and examined daily under a dissecting microscope. Hyphae observed emerging from larval bodies and growing into the agar were excised with a sterile razor blade and plated onto a fresh plate with selection as before. Isolates were purified three times by hyphal tipping and then maintained at room temperature on PDA.

### 2.3. Pythium Temperature Growth Tolerance Assays

To test the extent of the growth of the *Pythium* cultures at varying temperatures, agar wedges containing hyphae at the edges of actively growing cultures were cut from a PDA culture and used to inoculate the centers of fresh PDA plates. In triplicate, plates were incubated at 20 °C, 26 °C, and 37 °C for 4 days. Relative growth was assessed by measuring colony diameter on each plate.

### 2.4. DNA Extraction, PCR, and Sequencing

To extract DNA from the *Pythium* and *Trichoderma* isolates, 100 mm PDA plates were overlayed with a cellophane disk, which allowed the fungi to access nutrients from the medium but not to penetrate the agar with their hyphae. Plates were then inoculated on top of this cellophane layer by placing a small wedge of agar containing the hyphae of the desired isolate. Plates were allowed to grow at room temperature for 4–7 days until hyphae had spread to cover the cellophane disk, after which point the hyphae were scraped off the surface of the cellophane and placed into sterile 2 mL locking-cap tubes, each containing a single sterile glass bead. Tubes containing tissue were then snap-frozen in liquid nitrogen and lyophilized. The lyophilized tissue samples were then lysed in a bead beater (MP Fast-Prep 24, 4.0 m/s for 60 s, MP Biomedicals, Santa Ana, CA, USA). DNA was extracted from the powdered tissue using a Plant/Fungi DNA Isolation Kit (Norgen Biotek, Thorold, ON, CA), and quality was assessed using a nanophotometer (Implen, model NP80, Westlake Village, CA, USA). Extracted DNA was used as a template to amplify the ITS/LSU and COX1 regions from the *Pythium* isolate and the ITS/LSU, TEF1a, and RPB2 regions from the *Trichoderma* isolates using the primer sets UN-up18S42/UN-lo28S1220 (ITS/LSU), OomCoxI-Levup/Fm85mod (COX1), EF1-728F/TEF1LLErev (TEF1), and fRPB2-5F/fRPB2-7cr (RPB2) [39,40]. Taq 5× Master Mix (New England Biolabs, Ipswich, MA, USA) was used for all PCR reactions according to the manufacturer’s instructions, and amplification was confirmed by gel electrophoresis. Polymerase Chain Reaction (PCR) reactions were purified using the GeneJET PCR Purification Kit (Thermo Scientific, Waltham, MA, USA) and sequenced by Sanger sequencing (Pythium COX1, Trichoderma ITS/LSU) or Oxford Nanopore sequencing (Pythium ITS/LSU, Trichoderma TEF1 and RPB2), using the same primers as for the PCR by Eton Biosciences (San Diego, CA, USA) and Plasmidsaurus (Eugene, OR, USA), respectively.

### 2.5. Generating Phylogenies

Following sequencing, the data were downloaded and manually assessed in Geneious Prime (Biomatters, Inc., Boston, MA, USA) (Geneious Prime 2023.2, https://www.geneious.com, accessed on 6 June 2023). The ends of forward and reverse sequences for Sanger-sequenced samples were trimmed with an error probability limit of 0.05, and read directions were set. Sequences were then aligned using Geneious Alignment with the alignment type set to global alignment with free end gaps and a 65% similarity (5.0/−4.0) cost matrix, and consensus sequences were generated using the highest quality thresholds (60%), assigning the quality to total and calling Sanger heterozygotes >50%. Consensus DNA sequences were then exported from Geneious Prime and, along with previously published barcoding data for each locus for closely related *Pythium* or *Trichoderma* isolates, respectively, were aligned using Multiple Alignment using fast Fourier transform (MAFFT) v. 7.520 with default settings (a gap opening penalty of 1.53, an offset of 0.0, and a maximum number of iterative refinements of 0). Following alignment, the sequences were trimmed using Gblocks with the minimum block length set to 5 [41]. Trimmed sequence alignments for each locus were concatenated using the concat function in the Seqkit program for *Pythium* and *Trichoderma* sequences, respectively, and concatenated alignments were used to construct and test maximum likelihood trees using Randomized Axelerated Maximum Likelihood (RAxML) [42] with bootstrap values set to 2000 replicates. Trees were visualized in R Studio using the GGtree package [43]. Branch support values above 70 were displayed on trees. For the *Trichoderma* TEF1a and RPB2 sequences and the Pythium ITS/LSU sequence, PCR samples were sequenced using Oxford Nanopore long-read sequencing (Plasmidsaurus), and contigs were used directly in downstream sequence alignment and tree generation after assessing sequence quality via Basic Local Alignment Search Tool (BLAST) alignment.

### 2.6. Microscopy

To image *Pythium* zoospores bound to the surfaces of the infected mosquito larvae, individual larvae were first placed into empty 60 mm Petri dishes and washed with sterile distilled water. This water was then removed, and several drops of lactophenol cotton blue (Thermo Scientific, Waltham, MA, USA) were applied directly to the larvae and allowed to sit for several minutes before being removed with several more washes with sterile distilled water. Whole stained larvae were then imaged using a Zeiss Stemi 305 dissecting microscope (Zeiss Group International, Jena, Germany). For higher-magnification imaging of *Pythium* hyphae and other morphological structures, small amounts of actively growing cultures were cut out of Petri dishes, stained with lactophenol cotton blue, mounted on glass slides, and cover slipped, or actively growing hyphae from *Pythium* cultures were used to inoculate thin layers of PDA poured directly onto glass slides and incubated in a humid Tupperware container overnight before lactophenol blue staining. In both cases, after staining, the samples were cover-slipped and imaged at either 40× or 100× objective magnification using a Motic BA310E compound microscope with an attached Moticam X3 (Microscope Central, Feasterville, PA, USA) and a Keyence BZ-X800 fluorescence microscope (Keyence, Osaka, Japan) using bright-field settings. For *Trichoderma* microscopy, hyphae and spores were collected from the plate cultures using a toothpick and suspended in water. Then, 5–10 µL of this solution was placed on a microscope slide, and 5 µL of lactophenol blue was added to the drop to stain the fungal tissue. The slides were imaged as above using a Keyence BZ-X800 microscope. To obtain scanning electron microscopy images, mosquito larvae were immersed in 4% paraformaldehyde and 2.5% glutaraldehyde in a cacodylate buffer (Sigma Aldrich, St. Louis, MO, USA) overnight at 4 °C. The fixed larvae were washed with a 0.1 M cacodylate buffer three times, followed by 2% buffered osmium tetroxide (Sigma Aldrich, St. Louis, MO, USA). The post-fixed larvae were water-washed and dehydrated in a graded ethanol series of 25% through 100% with increasing concentrations of 5% to 10%. Following ethanol dehydration, the larvae were subjected to critical-point drying, including overnight in stasis mode (Autosamdri-815; Tousimis, Rockville, MD, USA). The dried larvae were mounted on carbon adhesive tabs on aluminum specimen mounts and gold–palladium sputter-coated. The specimens were examined using secondary electrons (SE) on a field-emission SEM (SU-5000; Hitachi High Technologies America, Schaumburg, IL, USA).

## 3. Results

### 3.1. Sampling Sites and Macroscopic and Microscopic Characterizations of Infected Mosquitoes

**Figure 1:**
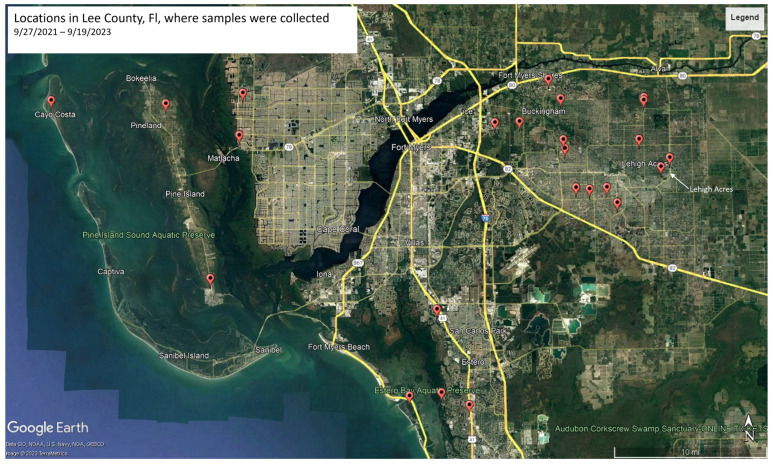
Geolocation map of *Pythium thermoculicivorax*-infected larval collections throughout Lee County, FL.

From late September until early November 2021, significant infection and mortality of mosquito larvae at eight different sites, some of which were sampled twice, within Lee County, Florida, were noted (Figure 1, Supplementary Table S1). Infection appeared to be >80%, with infected larvae including species visually identified as *Culex nigripalpus* (Theobald, 1901), *Psorophora columbiae* (Dyar & Knab, 1906), and *Aedes taeniorhynchus* (Wiedemann, 1821). Similarly, from early May until early December 2022, infected mosquito larvae were seen at various sites within Lee County that included the above-listed species, as well as infected *Culex interrogator* (Dyar & Knab, 1906) and *Culex quinquefasciatus* (Say, 1823) (Supplementary Table S1). Mosquito larvae were collected from three sites (in 2022), all containing infected *Ps. columbiae* larvae and further characterized as detailed below. Enzootic infections of mosquito larvae corresponding to *Cx. quinquefasciatus*, *Ae. taeniorhynchus*, *Aedes albopictus* (Skuse, 1894), and *Ps. columbiae* were also noted at various Lee County sites in May and September 2023.

**Figure 2:**
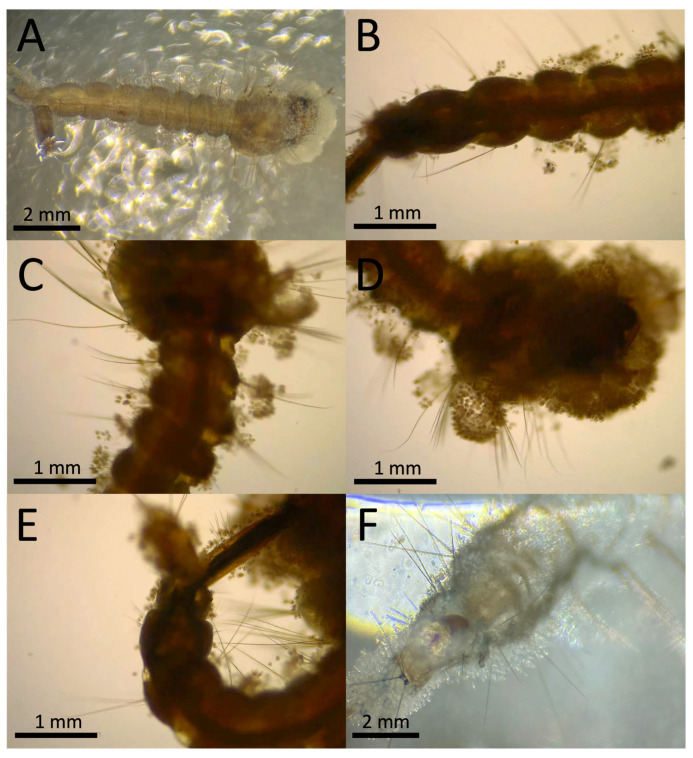
Representative images (**A**–**E**) of heavily infected *Culex quinquefasciatus* larva. (**F**) Heavy infection on empty larval exuvia.

**Figure 3:**
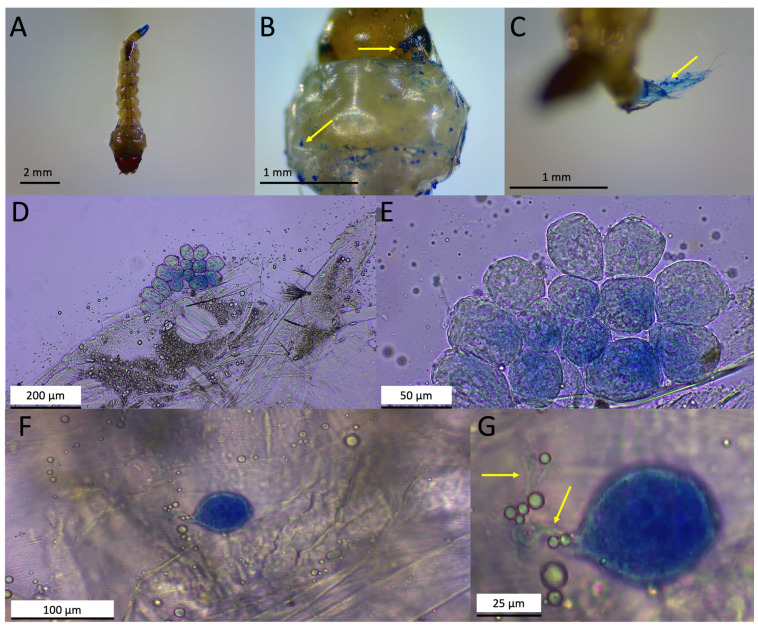
Representative images of infected mosquito larvae stained with lactophenol cotton blue dye and imaged using a dissecting microscope (**A**–**C**) and a compound microscope (**D**–**G**). (**A**) A whole stained mosquito larva showing clusters of blue infection structures (potentially encysted zoospores) along the body. (**B**) A higher-magnification image of infection structures (arrows) clustered around the head of the larva. (**C**) A higher-magnification image of infection structures (arrows) clustered around the siphon of the larva. (**D**) Mosquito larvae were crushed onto a microscope slide and examined at 100× oil immersion using a compound microscope. Encysted zoospores are evident in a cluster along the body of the larva. (**E**) Higher-magnification image of encysted zoospores showing gross morphology. (**F**) A single isolated zoospore cyst. (**G**) Infection and attachment structures evident on an isolated cyst (arrows).

**Figure 4:**
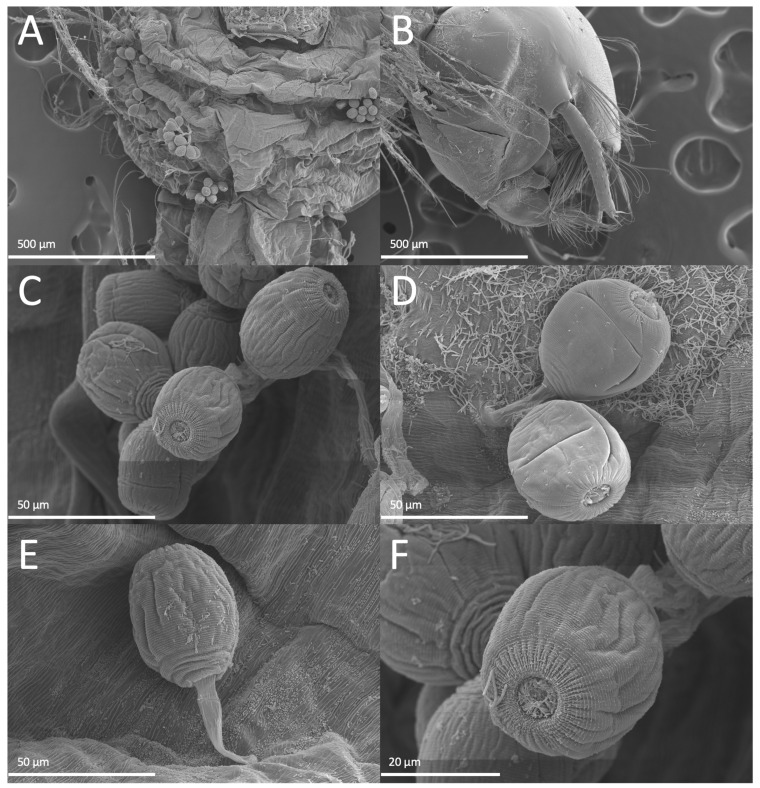
Representative images of scanning electron microscopy (SEM) analyses of infected mosquito larvae. (**A**) Encysted zoospores clustered along the underside of a mosquito larva head. (**B**) Microbial attachment and infection structures along the head. (**C**) Cluster of encysted zoospores appearing slightly oblong with shriveled lines along their sides and a distinct pore-like structure opposite to the attached end. (**D**) Two zoospore cysts attached (attachment structure visible) to the surface of the larva. (**E**) The profile of a single zoospore, showing the attachment/infection structure and the side morphology of the cyst. (**F**) A single zoospore cyst from a top view, showing the pore-like structure at the top of the cyst.

Sample I was collected on 26 August 2022 (Mercedes Court., Lehigh Acres, FL, USA); mosquito larvae from the sample site were visually inspected, with external microbial growth apparent throughout the bodies of most larvae collected (Figure 2). Clumps of cells could be seen on the head, thorax, and abdomen along various larval segments, as well as on seemingly indiscriminate hairs across the body. Compound microscopic visualization indicated that most samples appeared to display a similar microbial growth morphology on the larvae (24 were examined). To better observe the microbial growth, infected larvae were gently washed and stained with lactophenol blue (Figure 3). Whole stained mosquito larvae showed clusters of blue-stained cells dispersed throughout the body, with higher magnification often showing the strongest staining (and presumed microbial growth) at the head and along the siphon regions, including along the anal segment and pecten, with the latter region often showing the highest levels of infection, as seen by the intensity of dye staining (Figure 3A–C). Higher-resolution images of the cells on the larval cuticle revealed round to oval globular cells consistent with oogonia (Figure 3D,E). Apparent encysted zoospores with infection and attachment structures could also be seen (Figure 3E,F). In order to provide further morphological details on the infectious cells, the infected larvae were processed for scanning electron microscopy (SEM), as detailed in the Section 2. At lower magnifications, clumps of encysted zoospores could be readily identified on the mosquito larvae (Figure 4A,B). Higher-resolution imaging revealed slightly oblong encysted zoospores, with attachment and penetration tube-like structures evident (Figure 4C–E). A distinct pore-like, puckered, striated opening/structure could be seen at the opposite end of the attachment tubes (Figure 4F). For isolation, infected larvae were placed into a small divot cut into the center of a PDA plate amended with streptomycin and ampicillin and observed daily under a dissecting scope for the emergence of hyphae from the larval body into the agar. Tips of emerging hyphae were removed with a sterile scalpel blade and inoculated into the centers of fresh PDA plates with streptomycin and ampicillin to isolate cultures. This isolated strain was hyphal-tipped three successive times onto new PDA with streptomycin and ampicillin plates to obtain a pure culture, resulting in isolate LCMP-P1.

Samples II and III were collected on 15 November 2022 (Nursery Ln & Park Ln). Three infected mosquito larvae were chosen at random for the isolation of the infectious agent by hyphal tipping, as described above. Isolates were then purified via hyphal tipping and the successive passage (three times) of single colonies on PDA amended with streptomycin and ampicillin, resulting in isolates LCMP-T1, LCMP-T2, and LCMP-T3.

### 3.2. Phenotypic and Molecular Characterizations of LCMP-P1—Pythium thermoculicivorax

**Figure 5:**
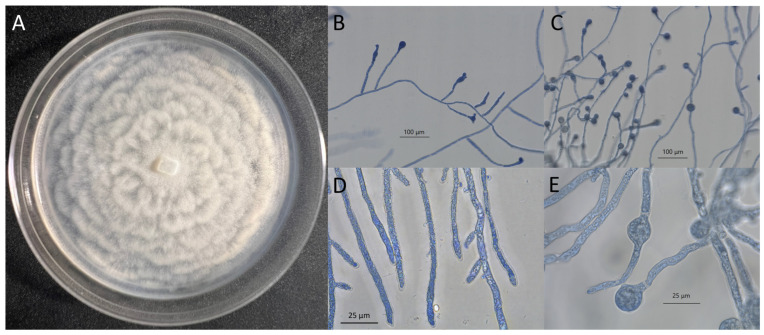
Isolate LCMP-P1 (*Pythium*): plate and hyphal morphology. (**A**) The plate morphology of the *Pythium* isolate grown on PDA at 24 °C for 7 days. (**B**) *Pythium* hyphae stained with lactophenol cotton blue showing sporangia forming on branched structures. (**C**) Mature sporangia forming from hyphae. (**D**) A close-up image of hyphae, showing their gross morphology. (**E**) A close-up image of sporangial structures.

**Figure 6:**
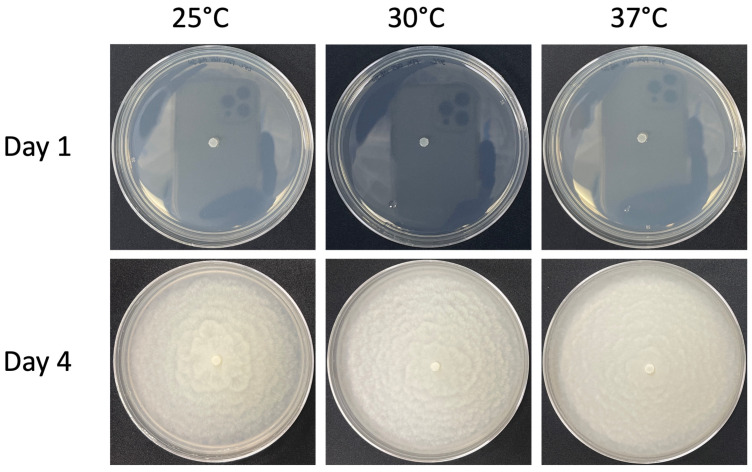
The temperature profile of Isolate LCMP-P1 (*Pythium*) grown on PDA plates. Isolate LCMP-P1 was inoculated into the centers of PDA plates in triplicate, grown at 3 different temperatures (25 °C, 30 °C, and 37 °C) and allowed to grow for 4 days, with the plates photographed at 1 d and 4 d post inoculation.

LCMP-P1 showed robust vegetative growth on a variety of media including PDA (Figure 5A), SDA, and CZA at 24 °C. On PDA, colonies appeared white to off-white and flattened, with a wet appearance and crenelated edges (Figure 5A). This isolate was observed to grow at an average rate of 18.93 mm/day on PDA. An examination of hyphal growth after lactophenol cotton blue staining showed sporangia forming on branched structures and mature sporangia within and developing at the ends of growing hyphae (Figure 5B,C). Growing filamentous hyphal structures showed high levels of internal vesicles, typical of many *Pythium* species, as well as sporangial structures that were globose with plerotic oospores (Figure 5D,E). Thermal tolerance experiments in which the PDA plates were incubated at different temperatures revealed that the isolate was temperature-resistant, showing robust vegetative growth at 30 °C and 37 °C similar to that seen at 25 °C, although it was noted that the distinct crenelations seen at 24 °C appeared to be increasingly smoothened at the higher temperatures (Figure 6).

Characteristics: Sporangia globosa and subglobosa, intercalaria; terminalia, 15–25 µm diameter; zoospores not observed. Oogonia globosa, intercalaria; terminalia, 16–20 µm diameter. Mycelium hyaline, moderately branched, 4–7 µm wide. Antheridia were sparsely noted in association with oogonia and took the form of short sections of hyphae which branched from the main hyphal strand just before an oogonium and then fused with oogonia, sometimes with a swollen end at the attachment site.

**Figure 7:**
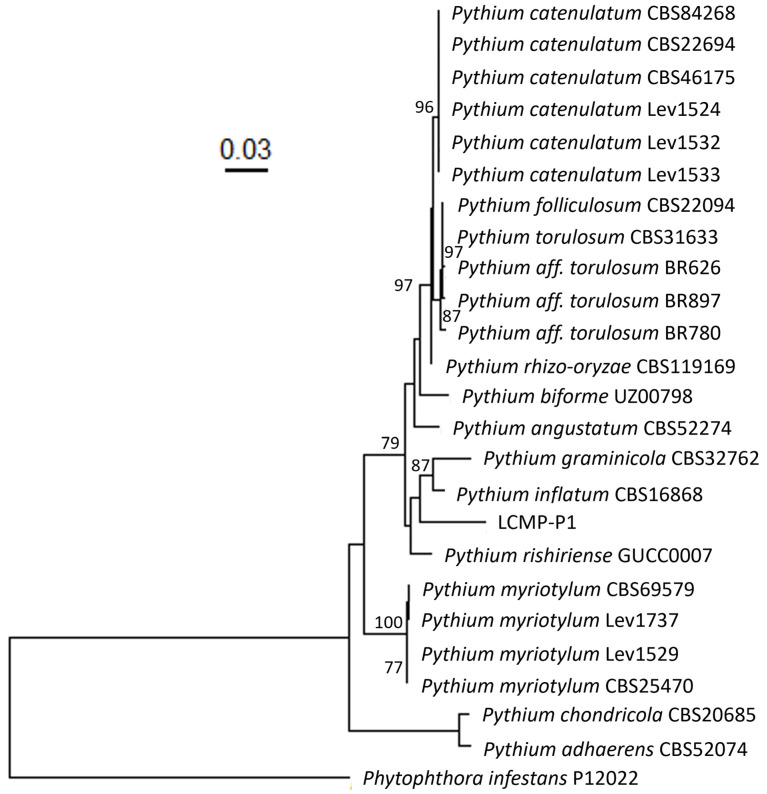
Maximum-likelihood phylogenetic tree of the combined ITS and COX1 loci of the *Pythium* isolate LCMP-P1 collected in this study and closely related to *Pythium* species. BLAST searches of this isolate showed sequence similarity to Clade B *Pythium* species. Sequences of closely related *Pythium* species within this clade, and several from different clades (*Pythium chondricola* CBS20685 and *Pythium adhaerens* CBS52074), were obtained from Genbank for both loci and used to generate phylogenetic trees. Phylogenies were generated using RAxML with 2000 bootstrap replicates. The *Pythium* isolate collected in this study appears to cluster most closely with *Pythium inflatum* CBS16868 but forms a distinct branch, suggesting high levels of sequence divergence between these two strains. *Phytophthora infestans* P12022 was used as an outgroup. Bootstrap support values >70 are displayed on the tree. Tip labels display species name and strain.

To establish the identity of this isolate, genomic DNA was extracted from growing mycelia, and the ITS and mitochondrial *COX1* loci were amplified and sequenced as detailed in the Section 2. A combined maximum-likelihood phylogenetic tree of 653 bp of the combined ITS and LSU (accession # PP262616) regions and 672 bp of the *COX1* (accession #PP107950) loci with sequences from related species confirmed the placement of the isolate within the *Pythium* genus, with *P. inflatum* CBS16868 being the closest neighbor in the tree and showing the highest sequence similarity in a BLAST search (95.17%), although the isolate was distinct from the latter (Figure 7).

Etymology: Due to its mosquito larval pathogen and thermal-tolerance phenotypes, the isolate is named *P. thermoculicovorax*.

### 3.3. Characterization of Isolates LCMP-T1, LCMP-T2, and LCMP-T3—Trichoderma sp.

**Figure 8:**
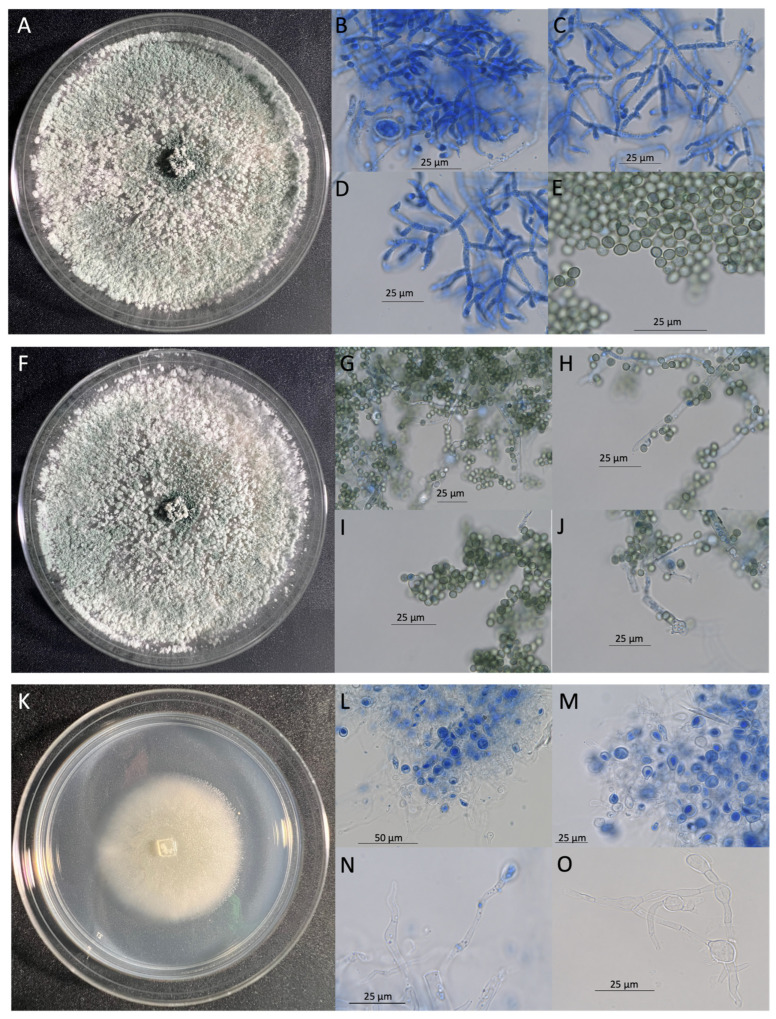
Plate and hyphal and conidial morphologies of Isolates LCMP-T2 (**A**–**E**), LCMP-T3 (**F**–**J**), and LCMP-T1 (**K**–**O**). (**A**,**F**,**K**) Plate morphologies on PDA at 24 °C 7 d. (**B**–**D**) Hyphae stained with lactophenol cotton blue, (**E**) green pigmented conidial spores, and (**G**) lactophenol blue-stained hyphae and spores. (**H**) Terminal hyphae showing septa and spores showing green pigmentation, (**I**) spores and potential conidiophore, (**J**) hyphae showing vesicles, potential conidiophore, and spores, (**L**) lactophenol blue-stained hyphae, (**M**) clumps of stained hyphae showing swollen terminal structures, (**N**) isolated hyphae showing septa and swollen terminal structures, and (**O**) hyphae showing swollen interior and terminal structures.

Isolates LCMP-T1, LCMP-T2, and LCMP-T3 all grew robustly on PDA, with the colonies of LCMP-T2 and LCMP-T3 turning green after 6–7 d of growth at 24 °C (Figure 8A–J), becoming a progressively darker shade of green after 7 d of growth. These isolates grew at average rates of 24.04 and 26.7 mm/day on PDA, respectively. Isolate LCMP-T1 was slower-growing, and the colony color was white after 7 d of growth (Figure 8K–O) and remained white to very pale green at 14 d. This isolate grew at an average rate of 23.46 mm/day on PDA. The lactophenol blue staining of growing vegetative cells of LCMP-T2 showed extensive branched hyphae and mycelia, with green conidial spores apparent (Figure 8B–E). Similarly, green conidial spores were apparent for LCMP-T3 (Figure 8G–J). The LCMP-T1 hyphae were hyaline and showed obvious septa and swollen terminal structures, with fewer spores that remained colorless (unless stained by the lactophenol blue) (Figure 8L–O).

**Figure 9:**
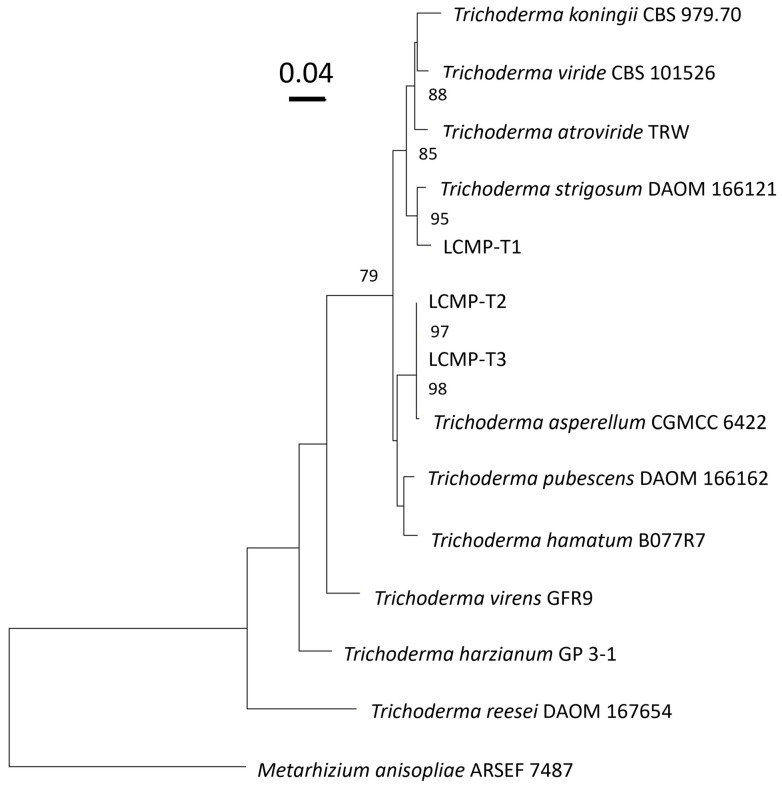
Maximum-likelihood phylogenetic tree of the ITS, TEF1a, and RPB2 sequences of Isolates LCMP-T1, LCMP-T2, and LCMP-T3 with closely related *Trichoderma* species. Sequences for closely related species at each of the three loci were obtained from Genbank. The phylogenetic tree was generated using RAxML with 2000 bootstrap replicates. In this tree, LCMP-T1 is seen to cluster most closely with *Trichoderma strigosum*, while isolates LCMP-T2 and LCMP-T3 formed a close cluster with *Trichoderma asperellum*. *Metarhizium anisopliae* ARSEF 7487 was used as an outgroup in the generation of this tree. Bootstrap support values >70 are displayed on the tree. Tip labels display species names and strains.

To establish the identities of these isolates, genomic DNA was extracted from growing vegetative cells, and the ITS (LCMP-T1 accession #PP125838, LCMP-T2 accession #PP125839, and LCMP-T3 accession #PP125840), *TEF1a* (LCMP-T1 accession #PP178665, LCMP-T2 accession #PP178663, and LCMP-T3 accession #PP178664), and *RBP2* (LCMP-T1 accession #PP178660, LCMP-T2 accession #PP178661, and LCMP-T3 accession #PP178662) loci were amplified and sequenced as detailed in the Section 2. Sequences were concatenated and used for the construction of a phylogenetic tree with closely related sequences as determined by BLAST searches of the NCBI database (Figure 9). These data showed that all three isolates clustered within *Trichoderma* sp., with isolates LCMP T2 and LCMP-T3 appearing closely related to each other and clustering with *T. asperellum* (99.84% identity) and Isolate LCMP-T1 clustering most closely with *T. strigosum* (98.19% identity).

### 3.4. P. thermoculcivorax Zoospores

Zoospores were not observed from standard culture media; however, after growth on V8 agar medium for 5–7 days, followed by the excision of a block of actively growing hyphae from the edge of a colony and its immersion in sterilized tap water for a period of time from 3 h to 3 days, a small number of zoospores were apparent (Supplementary Figure S1). These zoospores appeared oblong, with one end tapering slightly while the other end remained rounded; two central organellar structures were consistently noted in the center of each cell with biflagellate structures visible, and the cells were motile (Supplementary Videos S1–S3).

## 4. Discussion

Chemical pesticides continue to be the most widely used method for mosquito control, although alternatives, including natural products, gene targeting, sterile insect techniques, and biological agents such as mosquito pathogenic viruses, bacteria, and fungi, are actively being examined and sometimes even commercialized for mosquito control [15,44,45,46,47,48,49]. Methods for the facile isolation of microbial mosquito larval pathogens ranging from fungi to oomycetes have led to identification of several new species [50,51,52]. Of note, the use of many of these biological agents is both compatible with (though not necessarily needed) and capable of targeting insecticide-resistant insect populations, with little to no evidence of the emergence of resistance to the (fungal) agent [17,18,20,53,54,55,56].

The temperate and subtropical climate zones, coupled with high humidity and the presence of significant bodies of relatively static fresh water (swamps) in Florida (USA) have long been recognized as ideal breeding grounds for mosquitoes and the agents of (human and animal) disease they carry. Despite the abundance of mosquitoes found, there have been few studies examining mosquito enzootic infections in Florida. Here, we observed a series of natural enzootic outbreaks in several mosquito populations in Southwest Florida from 2021 to 2023. The mosquito larvae sampled from these sites had a large amount of microbial growth that could be readily seen by eye and confirmed via microscopy. Three sites containing infected *Ps. columbiae* larvae were used for the isolation of the infectious microbe via hyphal tipping on PDA plates, resulting in four strains designated as Isolates LCMP-P1, LCMP-T1, LCMP-T2, and LCMP-T3. Initial imaging of mosquitoes infected with Isolate LCMP-P1 was consistent with an oomycete as the infectious agent due to the observance of cells consistent with zoospore cysts. This was further confirmed via the SEM high-resolution morphological characterization of LCMP-P1 that showed clusters of encysted zoospores throughout the infected mosquito larvae, although they were concentrated in the head and siphon regions. The encysted zoospores displayed a unique morphology, with radial striations and a pore distal to the attachment tube. To the best of our knowledge these are the first images of this sort of oomycete infection in mosquito larvae. LCMP-P1 grew rapidly in culture and formed characteristic hyphae and sporangia consistent with those seen for many *Pythium* species. The nucleotide sequences of the nuclear ITS and mitochondrial COX1 loci were determined and used to construct a concatenated phylogenetic tree, establishing the identity of the isolate within the *Pythium* genus. This isolate grouped most closely to *P. inflatum*; however, it formed a distinct branch from this taxon. *P. inflatum* has been implicated as a pathogen of a number of crop plants, including strawberry, corn, and soybean, and it has been isolated from soil and water samples in Africa and Korea [57,58,59,60]; however, a description of it as an invertebrate or mosquito larval pathogen has not been reported.

Although mainly characterized as plant pathogens [61,62,63], various oomycete (in the classic literature, referred to as water molds) strains are also known for their human/mammalian pathogenicity (e.g., *P. insidiosum*) [64,65,66] and, within this context, several oomycete mosquito (facultative) pathogens have also been described. *Lagenidium giganteum* was briefly registered by the US Environmental Protection Agency for mosquito larval control; however, reports of potential life-threatening cases in dogs derailed its use [28,67,68]. *Leptolegnia chapmanii* has been shown to be pathogenic to various mosquito larvae, with methods for its detection in infected *Aedes aegypti* (Linnaeus, 1762) larvae reported [30,51]. Various *Pythium* species as well as one *Saprolegnia* sp. have been characterized as mosquito larval pathogens from soil samples [69], and an earlier report of a *Pythium* isolate causing high mortality in field-collected larvae of *Aedes sierrensis* (Lynch-Arribalzaga, 1891) reported motile zoospores in the infective stage that were strongly chemotactic to wounds in mosquito larvae [22]. Comparative genomic and transcriptomic analyses of the mosquito larval oomycete pathogen *P. guiyangense* has yielded insights into infection mechanisms and its putative virulence and effector repertoire, the putative roles of chitinases and tyrosine kinases [36,70]. In addition, preliminary molecular studies identified the role of a ser/thr-protein (AGC-) kinase family member, including the PKA cAMP-dependent protein kinases, in vegetative growth, stress response, and virulence [71].

As mentioned previously, the mammalian pathogen *P. insidiosum* was also isolated from infected mosquito larvae, with the investigated isolate capable of growing at 37 °C [35]. Intriguingly, the *Pythium* isolate described herein was also thermotolerant, capable of almost equivalent growth at 25 °C, 30 °C, and 37 °C. Because of this property and due to its (ITS and COXI sequences) divergence from its nearest characterized relative, *P. inflatum*, originally isolated from vegetative debris [72] and considered a plant (corn, strawberry, and soybean) pathogen [73], we designated our isolate as *P. thermoculicivorax*. Subsequent sampling at two different sites of infected *Ps. columbiae* larvae resulted in the identification of three *Trichoderma* isolates (Isolates LCMP-T1-3). Phylogenetic analyses using three loci, namely, the ITS, *TEF1a*, and *RBP2* sequences, grouped Isolate LCMP-T1 as closely related to *T. strigosum*. Isolates LCMP-T2 and 3 were found to be closely related to their closest phylogenetic neighbor, *T. asperellum*. A number of *Trichoderma* species have been identified as insect pathogens [74], although thus far, this does not include *T. strigosum*, which has apparently been mainly isolated from soil as a potential plant-protective species [75,76,77]. Isolates of *T. asperellum* appear to be more diverse in their biology, with several characterized as displaying plant-protective qualities capable of offering protection against plant pathogenic fungi including *Fusarium*, *Botrytis*, and *Phellinus* sp., that cause various rot, wilt, and mildew diseases in agricultural crops and trees [78,79,80,81,82]. *Trichoderma asperellum* has been applied in conjunction with the insect pathogenic fungus *Beauveria bassiana* (Bals.-Criv, 1835) in attempts to protect maize from the Asian corn borer (*Ostrinia furnacalis* (Guenee, 1854)) [83], and its nematocidal potential has also been reported [84]. *Trichoderma asperellum* has also been implicated as an anopheline larvicide [85], and cell-wall-degrading extracts from the fungus have shown larvicidal activity against *Ae. aegypti* [86]. Our data highlight the potential diversity and importance of examining enzootic infections in target insect species that can lead to the discovery of novel pathogens that may have important impacts in natural insect populations as well as serve as resources or reservoirs for the isolation of potential (mosquito) biological control agents.

## Data Availability

All data are available publicly and/or are included in the manuscript and Supplementary Materials.

## Supplementary Materials

The following supporting information can be downloaded at https://www.mdpi.com/article/10.3390/jof10030199/s1, Figure S1: Zoospores imaged in culture; Table S1: Locations where infected larvae were noted. Table S2: Primers used to amplify loci from *Pythium* and *Trichoderma* isolates; Tables S3 and S4: Genbank IDs of species and loci used for generating phylogenic trees; Video S1: Motile zoospores; Video S2: Zoospore with flagella visible; Video S3: Zoospore with flagella visible.
