# Supplementary material for: Isolation of a Novel Pythium Species, P. thermoculicivorax, and Trichoderma sp. from Natural Enzootic Mosquito Larval Infections
Source: J Fungi (Basel). 2024 Mar 5;10(3):199. doi: 10.3390/jof10030199 (PMC10971675; doi:10.3390/jof10030199)
Supplement: Supplementary file 1 [file jof-10-00199-s001.zip › revised-Supporting Data.pptx]

## Slide 1
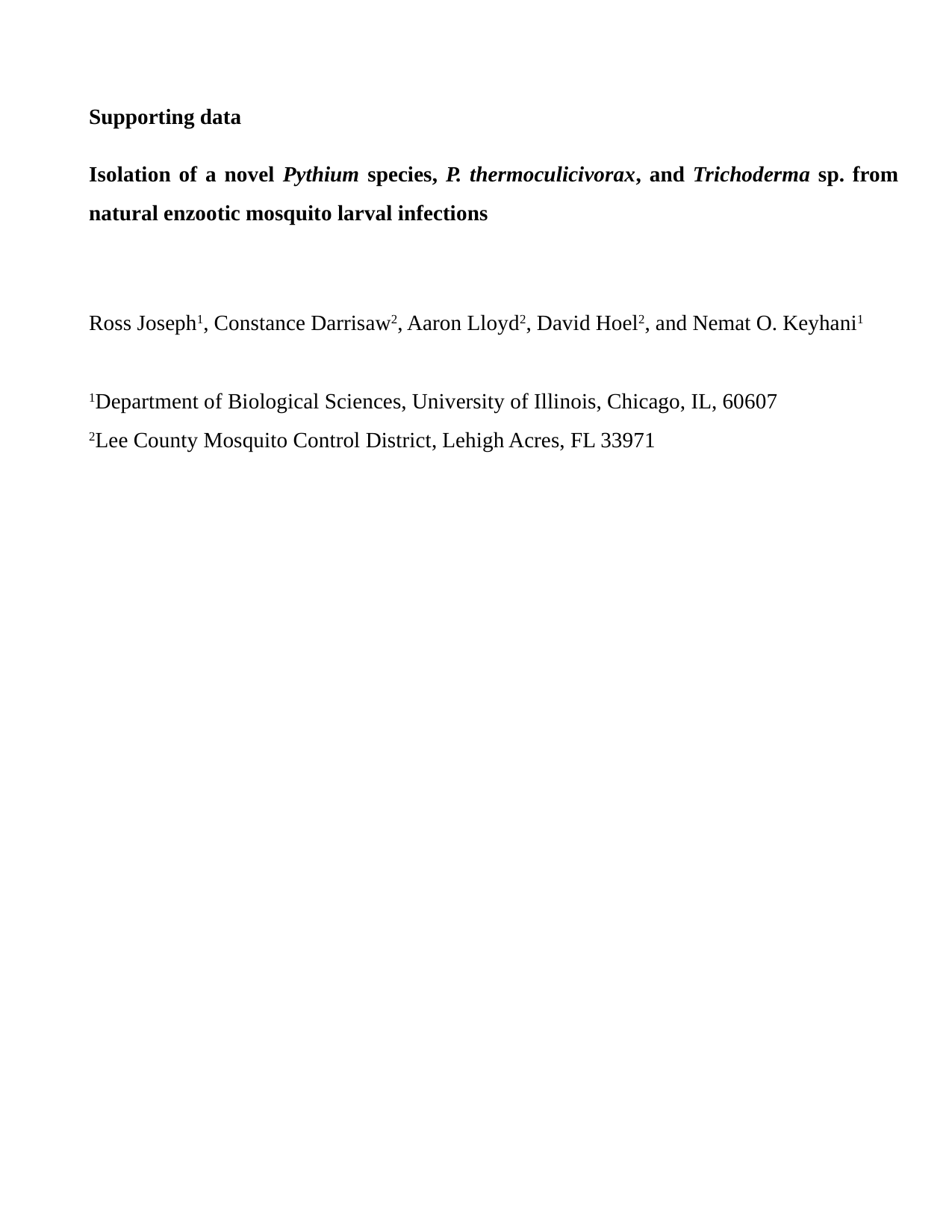

Supporting data
Isolation of a novel Pythium species, P. thermoculicivorax, and Trichoderma sp. from natural enzootic mosquito larval infections
Ross Joseph1, Constance Darrisaw2, Aaron Lloyd2, David Hoel2, and Nemat O. Keyhani1
1Department of Biological Sciences, University of Illinois, Chicago, IL, 60607
2Lee County Mosquito Control District, Lehigh Acres, FL 33971

## Slide 2
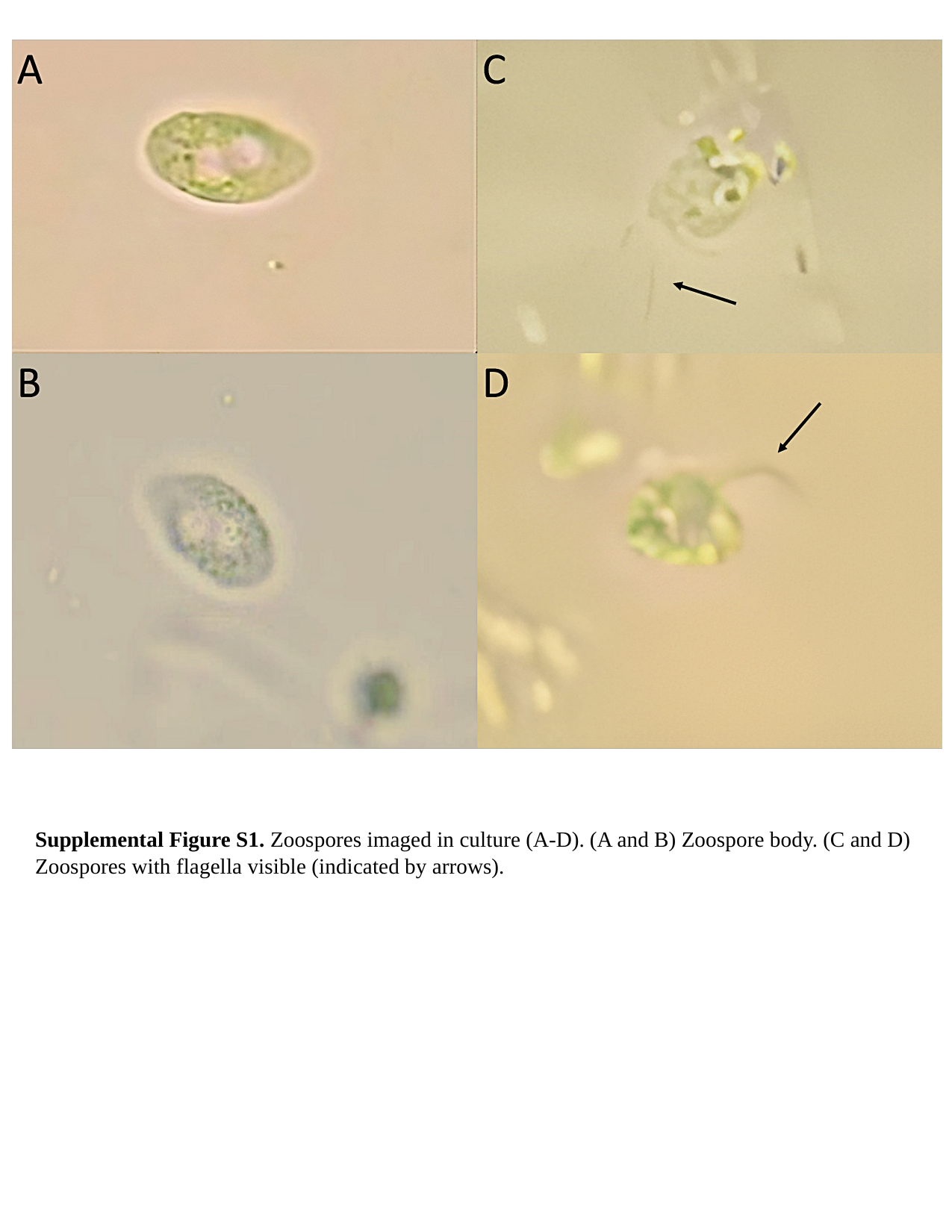

Supplemental Figure S1. Zoospores imaged in culture (A-D). (A and B) Zoospore body. (C and D) Zoospores with flagella visible (indicated by arrows).

## Slide 3
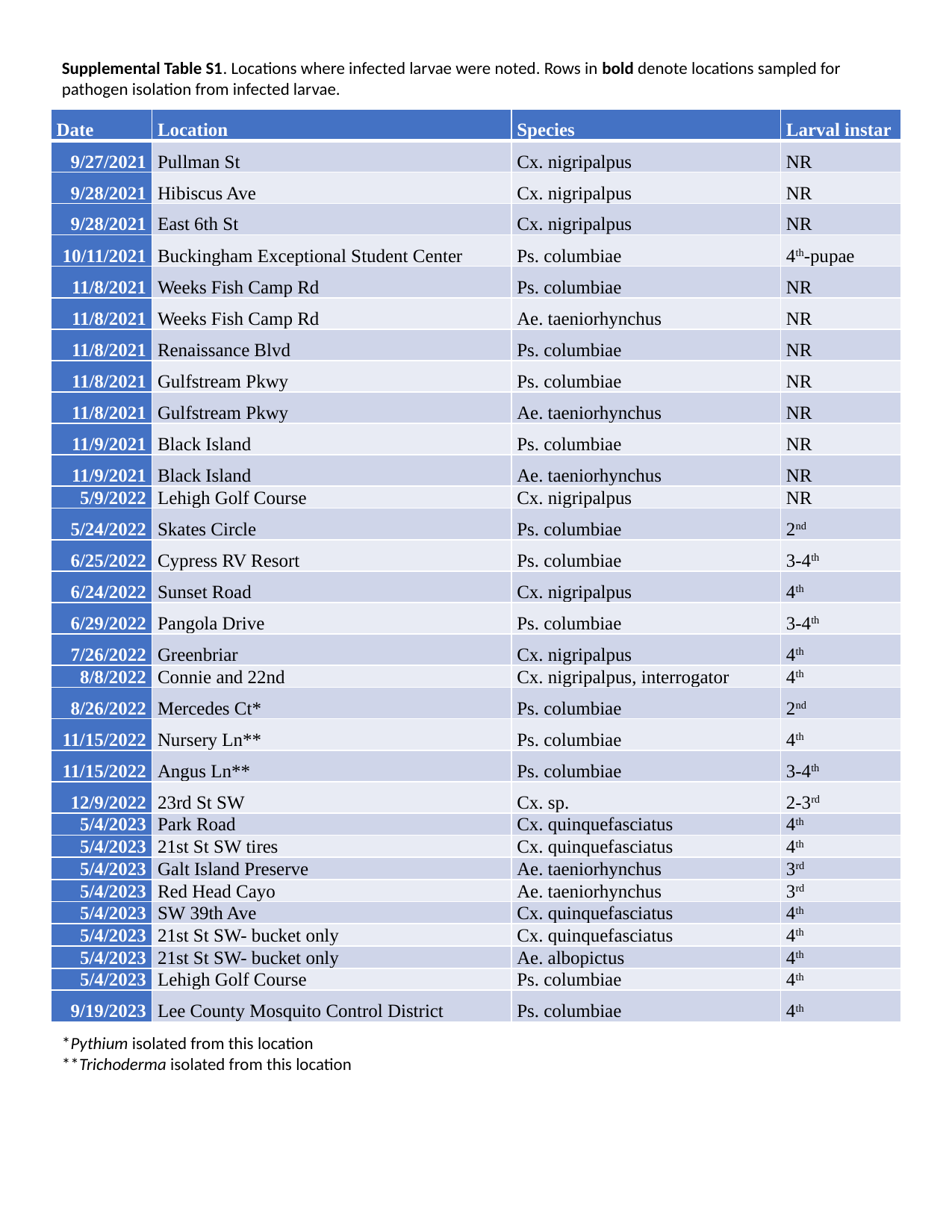

Supplemental Table S1. Locations where infected larvae were noted. Rows in bold denote locations sampled for pathogen isolation from infected larvae.
*Pythium isolated from this location
**Trichoderma isolated from this location
| Date | Location | Species | Larval instar |
| --- | --- | --- | --- |
| 9/27/2021 | Pullman St | Cx. nigripalpus | NR |
| 9/28/2021 | Hibiscus Ave | Cx. nigripalpus | NR |
| 9/28/2021 | East 6th St | Cx. nigripalpus | NR |
| 10/11/2021 | Buckingham Exceptional Student Center | Ps. columbiae | 4th-pupae |
| 11/8/2021 | Weeks Fish Camp Rd | Ps. columbiae | NR |
| 11/8/2021 | Weeks Fish Camp Rd | Ae. taeniorhynchus | NR |
| 11/8/2021 | Renaissance Blvd | Ps. columbiae | NR |
| 11/8/2021 | Gulfstream Pkwy | Ps. columbiae | NR |
| 11/8/2021 | Gulfstream Pkwy | Ae. taeniorhynchus | NR |
| 11/9/2021 | Black Island | Ps. columbiae | NR |
| 11/9/2021 | Black Island | Ae. taeniorhynchus | NR |
| 5/9/2022 | Lehigh Golf Course | Cx. nigripalpus | NR |
| 5/24/2022 | Skates Circle | Ps. columbiae | 2nd |
| 6/25/2022 | Cypress RV Resort | Ps. columbiae | 3-4th |
| 6/24/2022 | Sunset Road | Cx. nigripalpus | 4th |
| 6/29/2022 | Pangola Drive | Ps. columbiae | 3-4th |
| 7/26/2022 | Greenbriar | Cx. nigripalpus | 4th |
| 8/8/2022 | Connie and 22nd | Cx. nigripalpus, interrogator | 4th |
| 8/26/2022 | Mercedes Ct\* | Ps. columbiae | 2nd |
| 11/15/2022 | Nursery Ln\*\* | Ps. columbiae | 4th |
| 11/15/2022 | Angus Ln\*\* | Ps. columbiae | 3-4th |
| 12/9/2022 | 23rd St SW | Cx. sp. | 2-3rd |
| 5/4/2023 | Park Road | Cx. quinquefasciatus | 4th |
| 5/4/2023 | 21st St SW tires | Cx. quinquefasciatus | 4th |
| 5/4/2023 | Galt Island Preserve | Ae. taeniorhynchus | 3rd |
| 5/4/2023 | Red Head Cayo | Ae. taeniorhynchus | 3rd |
| 5/4/2023 | SW 39th Ave | Cx. quinquefasciatus | 4th |
| 5/4/2023 | 21st St SW- bucket only | Cx. quinquefasciatus | 4th |
| 5/4/2023 | 21st St SW- bucket only | Ae. albopictus | 4th |
| 5/4/2023 | Lehigh Golf Course | Ps. columbiae | 4th |
| 9/19/2023 | Lee County Mosquito Control District | Ps. columbiae | 4th |

## Slide 4
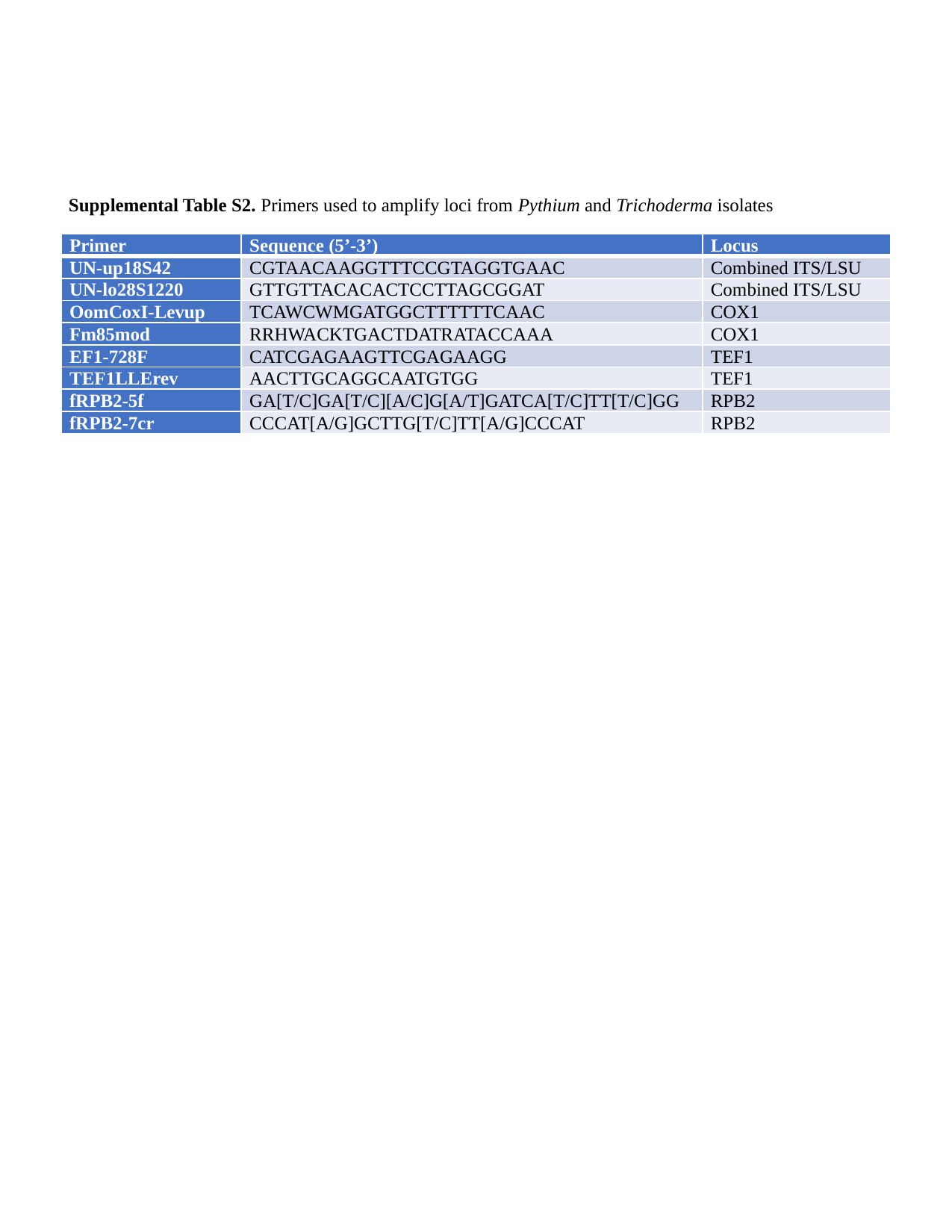

Supplemental Table S2. Primers used to amplify loci from Pythium and Trichoderma isolates
| Primer | Sequence (5’-3’) | Locus |
| --- | --- | --- |
| UN-up18S42 | CGTAACAAGGTTTCCGTAGGTGAAC | Combined ITS/LSU |
| UN-lo28S1220 | GTTGTTACACACTCCTTAGCGGAT | Combined ITS/LSU |
| OomCoxI-Levup | TCAWCWMGATGGCTTTTTTCAAC | COX1 |
| Fm85mod | RRHWACKTGACTDATRATACCAAA | COX1 |
| EF1-728F | CATCGAGAAGTTCGAGAAGG | TEF1 |
| TEF1LLErev | AACTTGCAGGCAATGTGG | TEF1 |
| fRPB2-5f | GA[T/C]GA[T/C][A/C]G[A/T]GATCA[T/C]TT[T/C]GG | RPB2 |
| fRPB2-7cr | CCCAT[A/G]GCTTG[T/C]TT[A/G]CCCAT | RPB2 |

## Slide 5
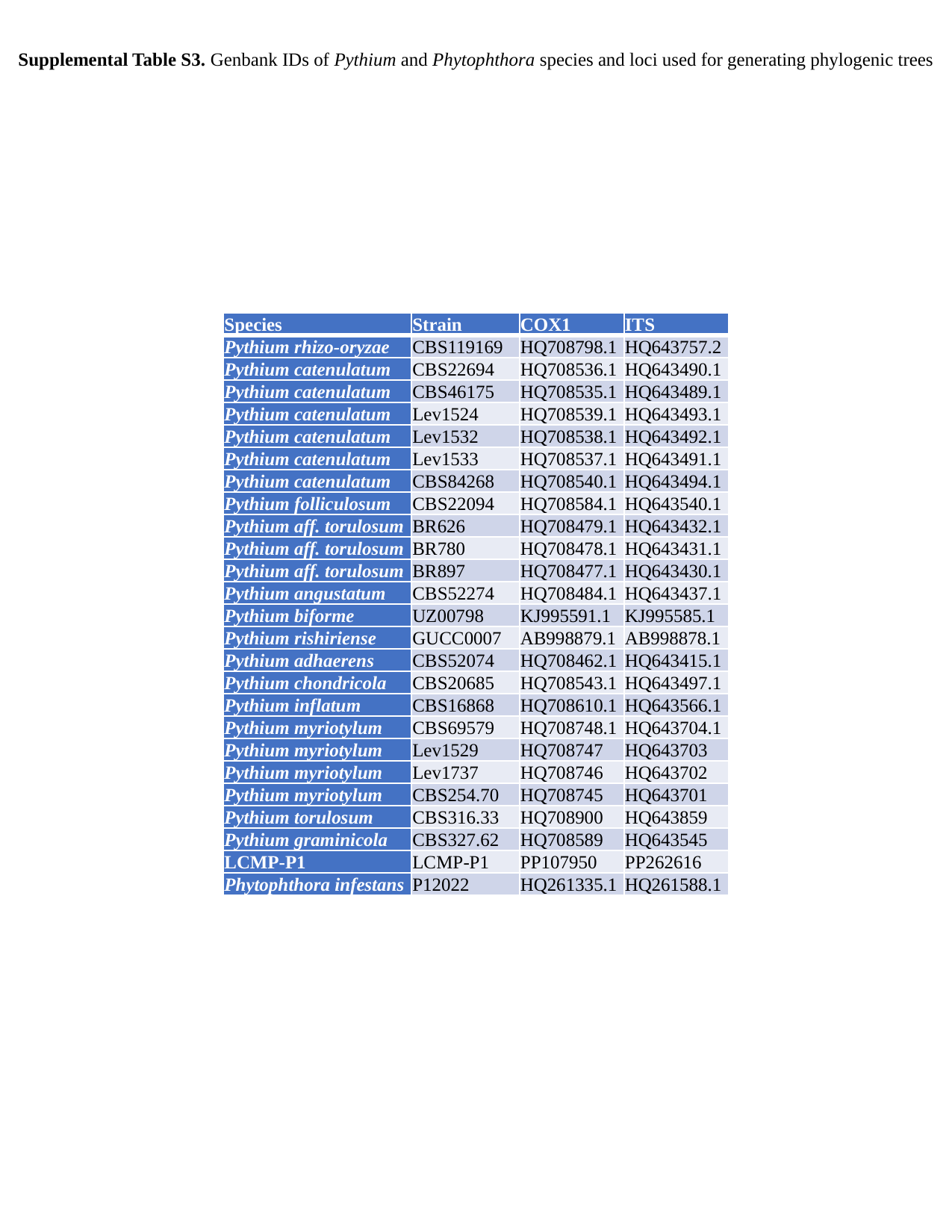

Supplemental Table S3. Genbank IDs of Pythium and Phytophthora species and loci used for generating phylogenic trees
| Species | Strain | COX1 | ITS |
| --- | --- | --- | --- |
| Pythium rhizo-oryzae | CBS119169 | HQ708798.1 | HQ643757.2 |
| Pythium catenulatum | CBS22694 | HQ708536.1 | HQ643490.1 |
| Pythium catenulatum | CBS46175 | HQ708535.1 | HQ643489.1 |
| Pythium catenulatum | Lev1524 | HQ708539.1 | HQ643493.1 |
| Pythium catenulatum | Lev1532 | HQ708538.1 | HQ643492.1 |
| Pythium catenulatum | Lev1533 | HQ708537.1 | HQ643491.1 |
| Pythium catenulatum | CBS84268 | HQ708540.1 | HQ643494.1 |
| Pythium folliculosum | CBS22094 | HQ708584.1 | HQ643540.1 |
| Pythium aff. torulosum | BR626 | HQ708479.1 | HQ643432.1 |
| Pythium aff. torulosum | BR780 | HQ708478.1 | HQ643431.1 |
| Pythium aff. torulosum | BR897 | HQ708477.1 | HQ643430.1 |
| Pythium angustatum | CBS52274 | HQ708484.1 | HQ643437.1 |
| Pythium biforme | UZ00798 | KJ995591.1 | KJ995585.1 |
| Pythium rishiriense | GUCC0007 | AB998879.1 | AB998878.1 |
| Pythium adhaerens | CBS52074 | HQ708462.1 | HQ643415.1 |
| Pythium chondricola | CBS20685 | HQ708543.1 | HQ643497.1 |
| Pythium inflatum | CBS16868 | HQ708610.1 | HQ643566.1 |
| Pythium myriotylum | CBS69579 | HQ708748.1 | HQ643704.1 |
| Pythium myriotylum | Lev1529 | HQ708747 | HQ643703 |
| Pythium myriotylum | Lev1737 | HQ708746 | HQ643702 |
| Pythium myriotylum | CBS254.70 | HQ708745 | HQ643701 |
| Pythium torulosum | CBS316.33 | HQ708900 | HQ643859 |
| Pythium graminicola | CBS327.62 | HQ708589 | HQ643545 |
| LCMP-P1 | LCMP-P1 | PP107950 | PP262616 |
| Phytophthora infestans | P12022 | HQ261335.1 | HQ261588.1 |

## Slide 6
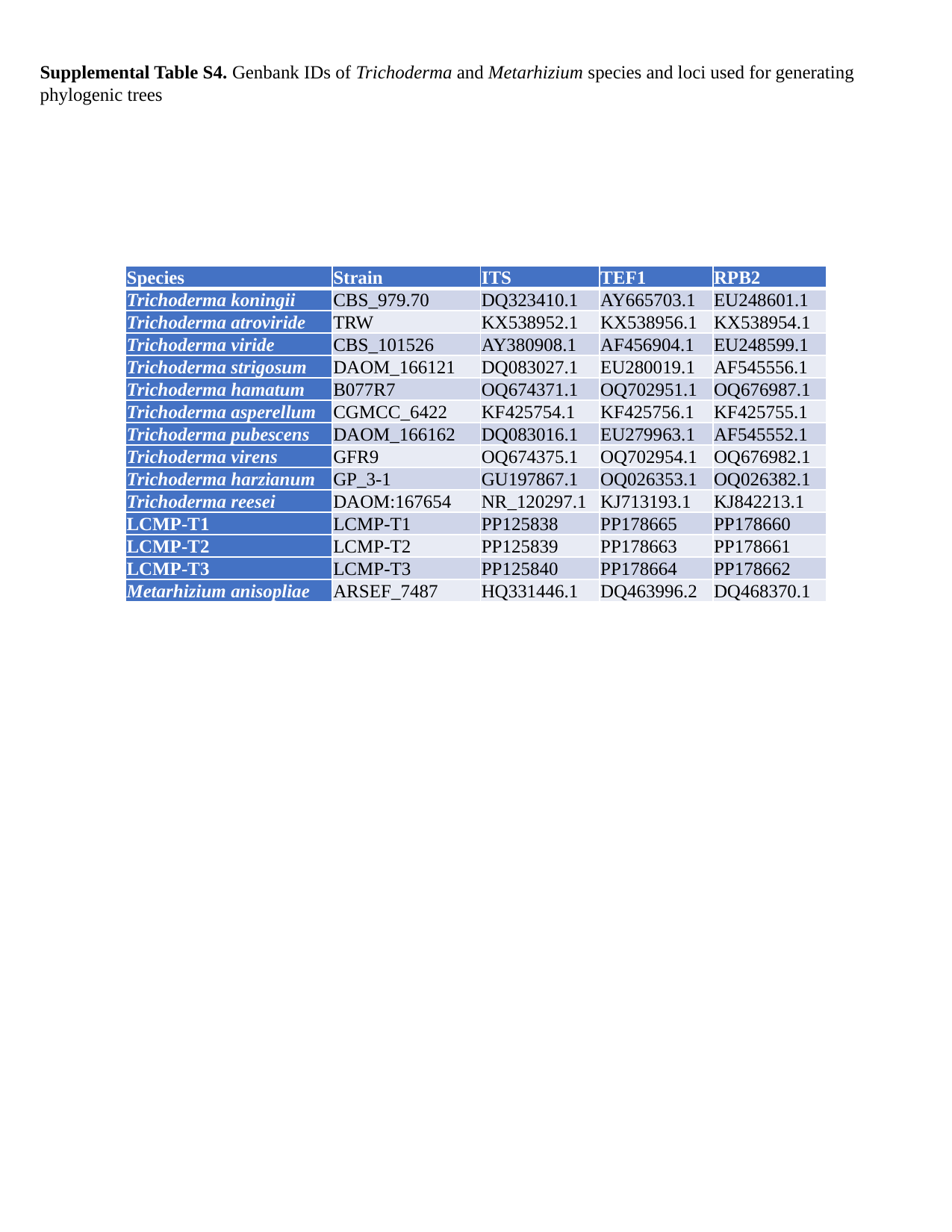

Supplemental Table S4. Genbank IDs of Trichoderma and Metarhizium species and loci used for generating phylogenic trees
| Species | Strain | ITS | TEF1 | RPB2 |
| --- | --- | --- | --- | --- |
| Trichoderma koningii | CBS\_979.70 | DQ323410.1 | AY665703.1 | EU248601.1 |
| Trichoderma atroviride | TRW | KX538952.1 | KX538956.1 | KX538954.1 |
| Trichoderma viride | CBS\_101526 | AY380908.1 | AF456904.1 | EU248599.1 |
| Trichoderma strigosum | DAOM\_166121 | DQ083027.1 | EU280019.1 | AF545556.1 |
| Trichoderma hamatum | B077R7 | OQ674371.1 | OQ702951.1 | OQ676987.1 |
| Trichoderma asperellum | CGMCC\_6422 | KF425754.1 | KF425756.1 | KF425755.1 |
| Trichoderma pubescens | DAOM\_166162 | DQ083016.1 | EU279963.1 | AF545552.1 |
| Trichoderma virens | GFR9 | OQ674375.1 | OQ702954.1 | OQ676982.1 |
| Trichoderma harzianum | GP\_3-1 | GU197867.1 | OQ026353.1 | OQ026382.1 |
| Trichoderma reesei | DAOM:167654 | NR\_120297.1 | KJ713193.1 | KJ842213.1 |
| LCMP-T1 | LCMP-T1 | PP125838 | PP178665 | PP178660 |
| LCMP-T2 | LCMP-T2 | PP125839 | PP178663 | PP178661 |
| LCMP-T3 | LCMP-T3 | PP125840 | PP178664 | PP178662 |
| Metarhizium anisopliae | ARSEF\_7487 | HQ331446.1 | DQ463996.2 | DQ468370.1 |

## Slide 7
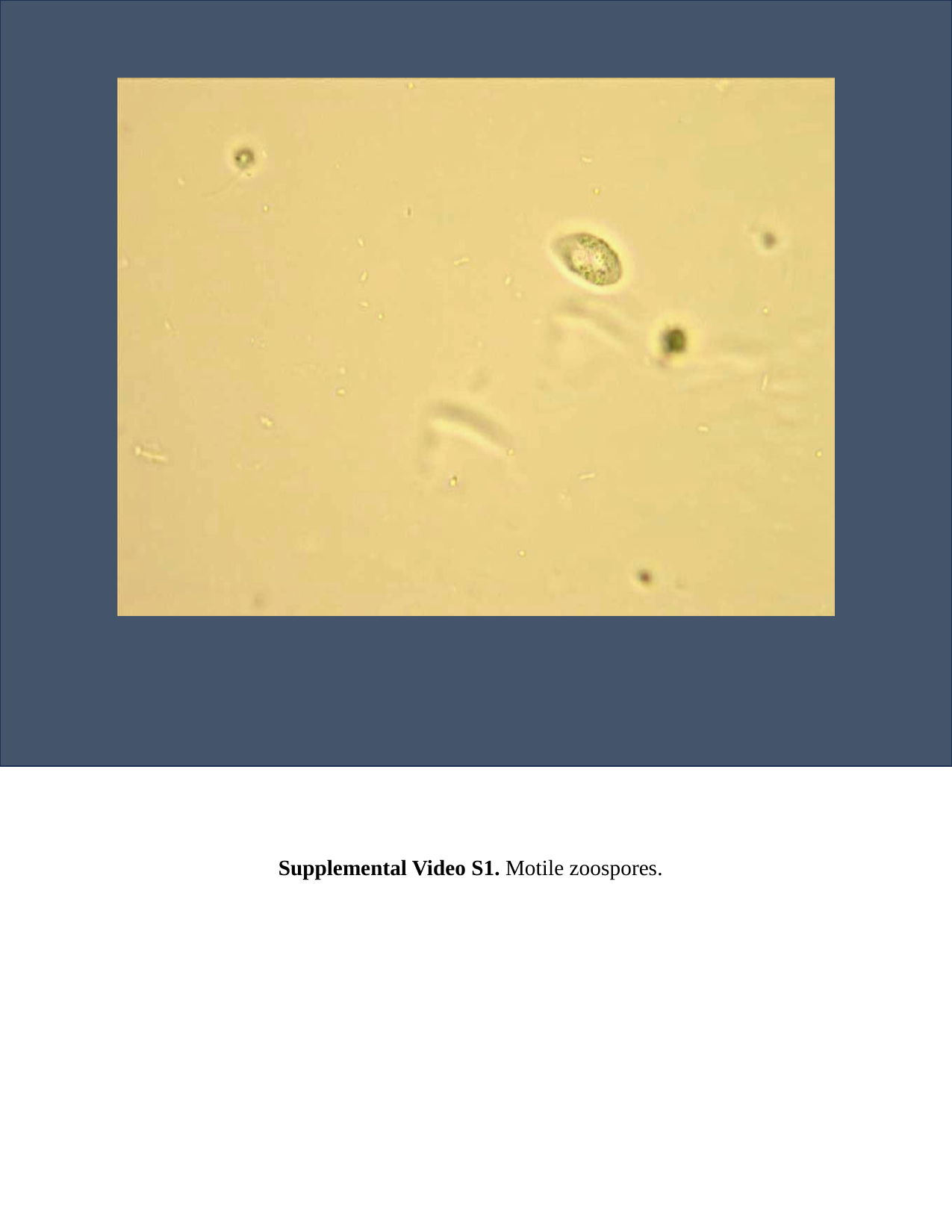

Supplemental Video S1. Motile zoospores.

## Slide 8
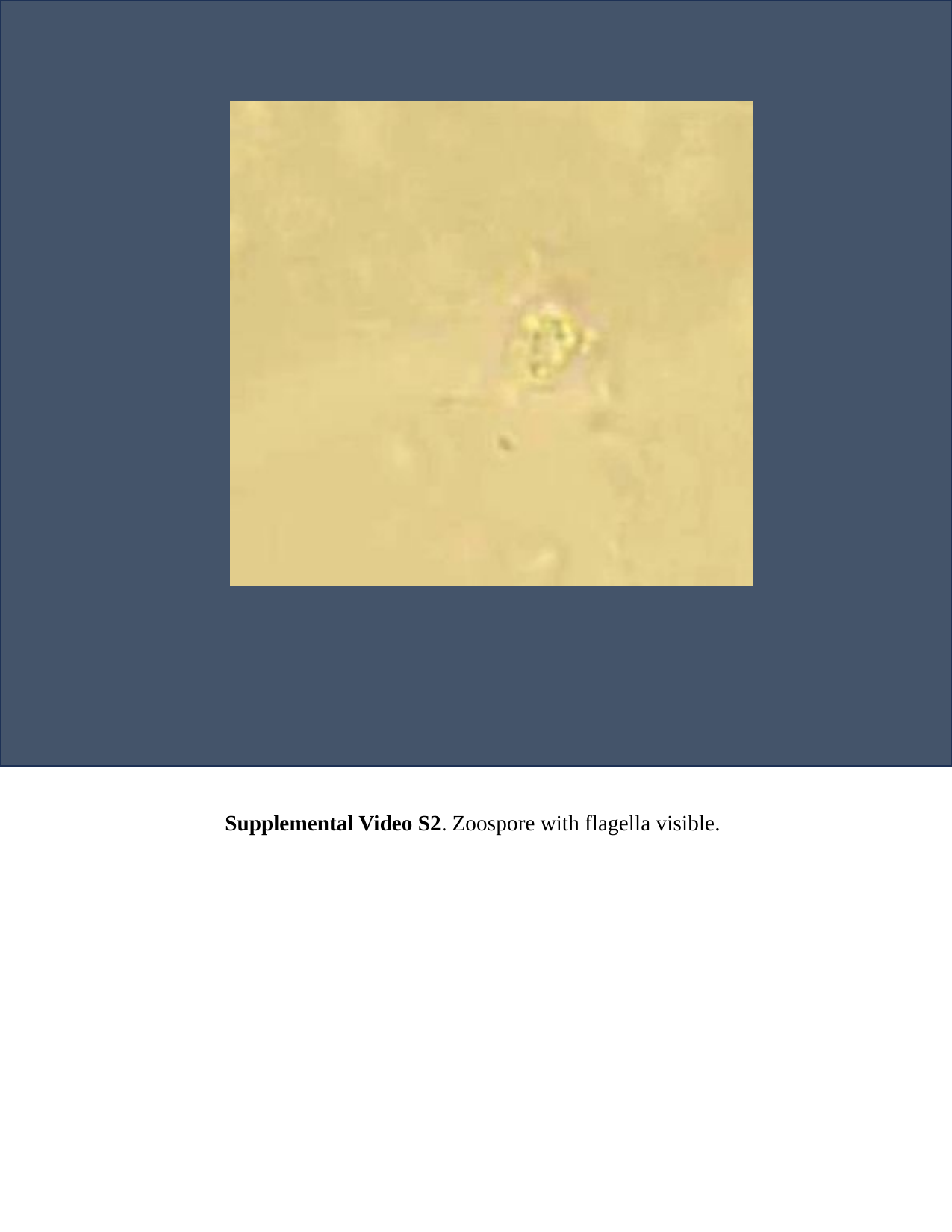

Supplemental Video S2. Zoospore with flagella visible.

## Slide 9
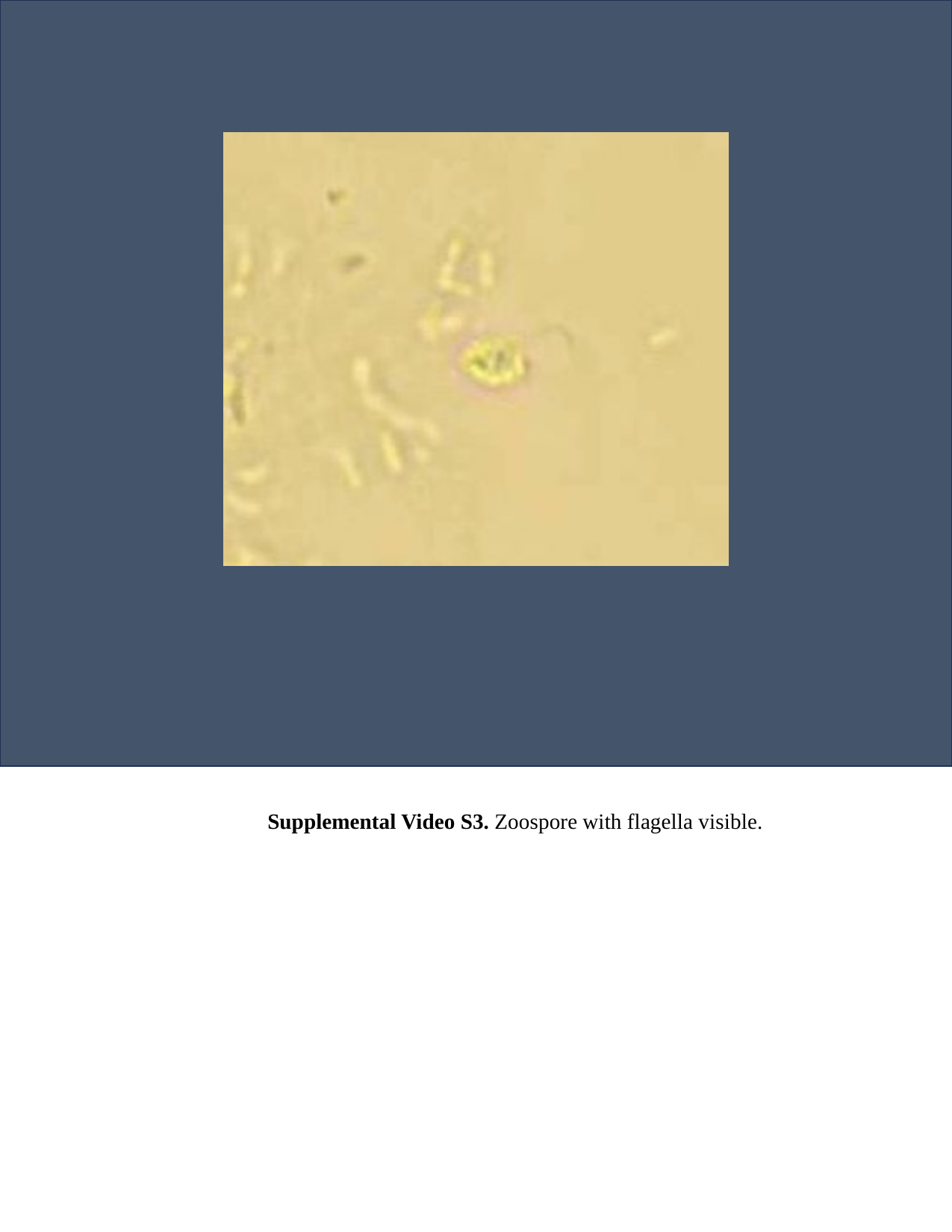

Supplemental Video S3. Zoospore with flagella visible.
